# Methodological recommendations for human microbiota-gut-brain axis research

**DOI:** 10.20517/mrr.2023.33

**Published:** 2023-10-17

**Authors:** Yangwenshan Ou, Clara Belzer, Hauke Smidt, Carolina de Weerth

**Affiliations:** ^1^Laboratory of Microbiology, Wageningen University & Research, P.O. Box 8033, 6700 EH Wageningen, The Netherlands.; ^2^Radboud university medical center, Donders Institute for Brain, Cognition and Behaviour, Department of Cognitive Neuroscience, P.O. Box 9010, 6500 GL Nijmegen, The Netherlands.

**Keywords:** Microbiota-gut-brain axis, gut microbiota, mental development and health, correlation, causation, confounders, statistical analyses

## Abstract

Observational studies have determined numerous correlations between sequence-based gut microbiota data and human mental traits. However, these associations are often inconsistent across studies. This inconsistency is one of the reasons that mechanistic validation studies of the observed correlations are lagging, making it difficult to establish causal associations. The absence of consistent study findings may partially be due to the lack of clear guidelines for identifying confounders of relations between complex microbial communities and mental conditions. Gut microbial complexity also impedes deciphering microbiota-host relations by using a single analytical approach. The aim of the current review is to help solve these problems by providing methodological recommendations for future human microbiota-gut-brain axis research on the selection of confounders, the use of integrative biostatistical methods, and the steps needed to translate correlative findings into causal conclusions.

## INTRODUCTION

In the past decades, scientists have found that germ-free, antibiotic-treated, or gnotobiotic rodents show substantial changes in brain physiology and behavior^[1]^. These findings were paramount in establishing the emerging role of the gut microbiota in brain development and mental health. In the following years, differences in microbial composition were frequently observed in case-control studies between neurotypical individuals and those with psychopathologies such as attention deficit hyperactivity disorder (ADHD), autism spectrum disorder (ASD), major depressive disorder (MDD), and generalized anxiety disorder (GAD)^[2-5]^. Recently, with a rise in the number of cohort studies, longitudinal relations between gut microbiota and host behavior have been reported more often^[6-10]^. Meanwhile, animal studies have served to discover several potential molecular mechanisms underlying observations in humans, including pathways related to aspects of immunity, the endocrine system, and the vagus nerve^[1,11,12]^. Specifically, the gut microbiota can generate a wide variety of metabolites, such as short-chain fatty acids and neurotransmitters. These neuroactive microbial metabolites can influence the gut barrier and dendritic cells to regulate immune function, as well as affect enterochromaffin cells to release gastrointestinal hormones. In addition, microbial signals can be detected by the vagus nerve in the form of microbiota-derived metabolites, or directly influence brain function via the vagus nerve as indicated by vagotomy studies. Collectively, these links between the gut and the brain are defined as the microbiota-gut-brain axis (MGBA)^[1,11]^.

Due to the wide use of DNA sequencing techniques in describing gut microbiota composition, a high number of correlations have been found between the microbiota and host observable traits (e.g., brain structure and function, and host behavior). However, these associations are mostly variable and inconsistent across observational studies, and lack follow-up validation^[2-10]^. The lack of consistency in findings also makes it more challenging to set up mechanistic validation studies aimed at establishing causal associations. And indeed, mechanistic validation studies on correlational findings fail to keep pace, largely hampering their biological interpretation and translation towards clinical applications.

The inconsistencies in correlational findings can be attributed to different approaches from microbiology, genomics, epidemiology, bioinformatics, statistics, and other fields^[13]^. To facilitate the replicability and reproducibility of human microbiome research, a multi-disciplinary working group has adapted and developed a checklist called Strengthening The Organization and Reporting of Microbiome Studies^[13]^. This checklist provides guidance on how to concisely and completely report microbiome findings, of which the selection of potential confounders is an important part. Confounders are variables that influence both predictors and outcomes, and that require proper identification before being selected for use in multivariate statistical analysis. It is not easy to identify confounders of relations between diverse and complex microbial communities and mental traits because of limited knowledge of these intricate systems. This complication makes examining relations along the MGBA a challenging endeavor. Next to finding adequate ways of identifying confounders, the currently used statistical analyses are in need of close inspection and improvement, as they also may be behind inconsistencies in correlational findings. To this end, it is necessary to not only adopt suitable analytical methods but also use them integratively.

In this review focused on human MGBA research, we present methodological considerations aimed at helping to move the field forward: on the selection of confounders, on statistical approaches, and on how to move from correlation to causal inferences.

## CHALLENGES IN SELECTING CONFOUNDERS

For observational studies aiming to infer potential causal relations, it remains a major concern to reduce the bias introduced by confounders^[14,15]^. It is not a simple endeavor to identify confounders in studies focused on associations between complex systems with numerous variables (e.g., the gut microbiota and host behavior), as knowledge about the relations between these variables is often insufficient and unavailable.

In the following paragraphs, we present several considerations for choosing confounders, with the goal of inspiring the field:

(1) It is common in this field to choose potential confounders by referring to what has been previously reported in the literature. The most frequently used confounders when exploring relations along the MGBA in community samples include age, sex, BMI (birth weight for infants), diet (breastfeeding for infants and children), antibiotic use, and gastrointestinal symptoms. In addition, alcohol consumption frequency has recently been identified as a microbiota-related confounding host variable by Vujkovic-Cvijin *et al.*, despite the fact that this variable has not received sufficient attention in the MGBA studies to date^[15]^. For infants and children, additional confounders may need to be carefully addressed, i.e., gestational duration, delivery mode, siblings, age, parental income, lifestyle, health conditions, and education level of parents (especially of primary caregiver). However, the current scarcity of MGBA research about microbiota-host links in specific fields (e.g., microbial relations to problem behavior and prosociality in community samples) means that very few references are available for confounder selection. Table 1 illustrates the variation in the use of confounders in published studies^[6-10,16-24]^. Furthermore, as the gut microbiota and host observable traits of mental development and health are sensitive to many variables (known *vs.* unknown; detectable *vs.* undetectable), it is nearly impossible to include all of them. To visualize such complex relations, a directed acyclic graph (DAG) can be helpful, as it provides insight into variables that have to be accounted for^[25]^. Criteria to identify such variables with the use of DAGs have been elaborated by Cinelli *et al.*^[26]^. In microbiota research, Eckermann *et al.* used a DAG to graphically describe potential confounders of the relation between gut microbiota and executive functions. This, in turn, provided a strong rationale for choosing the specific confounders for the analyses^[27]^.

**Table 1:** Commonly included confounders in human MGBA research focused on community samples

**Parameter**	**Design**	**Sample size; ages; country**	**Potential confounders**	**Year and ref.**
Cognition	Longitudinal	*N* = 89; ages = one and two years; USA	Age, sex, birth weight, delivery mode, siblings, breastfeeding, parental education level	2018^[6]^
	Longitudinal	*N* = 309; ages = three to six months and three years; USA	Age, sex, delivery mode, breastfeeding, antibiotic use, gestational duration, parental education level, parental ethnicity, family income	2019^[7]^
	Cross-sectional	*N* = 39; age = one year; USA	Age, sex, birth weight, delivery mode, siblings, breastfeeding, antibiotic use, gastrointestinal symptoms, gestational duration, parental education level, parental ethnicity, parental age at childbirth	2019^[20]^
	Cross-sectional	*N* = 46; age = three years; China	Age, sex, breastfeeding, parental age, parental education level	2021^[21]^
	Longitudinal	*N* = 260; ages = six weeks and one to three years; USA	Age, sex, delivery mode, breastfeeding, gestational duration, parental age, maternal education level, maternal smoking	2021^[22]^
	Longitudinal	*N* = 405; ages = one and two years; Canada	Age, sex, delivery mode, siblings, breastfeeding, antibiotic use, ear infection, maternal ethnicity, maternal weight, maternal antibiotic use, family income	2021^[8]^
	Longitudinal	*N* = 90; ages = birth to 60 months; Italy	Sex, delivery mode	2022^[23]^
Problem behavior	Longitudinal	*N* = 201; ages = one month to two years; Australia	Age, sex, delivery mode, siblings, breastfeeding, antibiotic use, gestational duration, pets	2020^[9]^
	Longitudinal	*N* = 260; ages = six weeks to three years; USA	Age, sex, delivery mode, breastfeeding, gestational duration, parental age, maternal education level, maternal smoking	2021^[22]^
	Longitudinal	*N* = 193; ages = one month to ten years; The Netherlands	Age, sex, birth weight, delivery mode, breastfeeding, solid food, antibiotic use	2022^[10]^
	Cross-sectional	*N* = 248; age = four years on average; Canada	Birth weight, delivery mode, antibiotic use, diet, maternal age, maternal education level, family income	2022^[24]^
	Cross-sectional	*N* = 1,784; age = ten years; Multi-country	Age, sex, BMI, antibiotic use, host genetics, country of origin, maternal education, technical factors related to microbial processing	2023^[16]^
Depression-relevant mental outcomes	Cross-sectional	Tested cohort *N* = 1,054; age = 51 years on average; Belgium Validated cohort*N* = 1,070; age = 45 years on average; The Netherlands	Age, sex, BMI, stool consistency, gastrointestinal symptoms, antidepressant use	2019^[17]^
	Longitudinal	*N* = 786; age = 65 to 69 years on average; UK	Age, sex, BMI, diet, antidepressant use, technical factors related to microbial processing	2021^[18]^
	Cross-sectional	*N* = 3,211; age = 50 years on average; Multi-country	Age, sex, BMI, education, ethnicity, physical activity, smoking, alcohol use, antibiotic use, proton-pump inhibitor use, gastrointestinal symptoms, diabetes	2022^[19]^

MGBA: Microbiota-gut-brain axis.

(2) When assessing a confounding effect, statistical significance is often determined based on a simple *P* value. The *P* value is used to decide whether to accept or reject the null hypothesis. Although widely adopted so far, more and more researchers have called for an end to simply using such a conventional and dichotomous way when declaring if an outcome rebuts or supports a hypothesis^[28]^. Instead of being overdependent on a *P* value, more attention should be given to a confidence interval (or a credible interval), which provides the range of plausible values of a relation^[29]^.

(3) Collinearity can happen when two or more variables are strongly interrelated. Although researchers are aware of this phenomenon, the degree of collinearity has not been frequently reported in previous microbiota studies. Including confounders with high collinearity levels can distort the interpretation of outcomes, and for this reason, it is advisable to pre-check and report collinearity.

(4) Presenting both crude relations without confounders and adjusted relations with confounders is a common practice in epidemiological research^[30-32]^. This provides information about how confounders influence associations and increases the interpretability of outcomes. For this reason, it is advisable to show both relations when studying microbiota-host links.

(5) As a good step forward, pre-registering considerations and methods that will be used for confounder selection (also in the exploration of the MGBA discussed later) on open science platforms are highly recommended. Study pre-registration strengthens the transparency, credibility, and scientific value of a study by reporting original data analysis plans, hence reducing the chances of *P*-hacking or data dredging, and of reporting chance findings.

In sum, there is no gold standard method for confounder selection and no consensus on the basis of which confounders have to be included in MGBA research. As a consequence, different studies often comprise a varied set of confounders, making comparisons and meta-analyses often hard to implement. Following the suggestions presented above can help improve the solidity and comparability of the results of this research field.

## EXPLORING THE MGBA THROUGH INTEGRATIVE ANALYTICAL APPROACHES

The gut microbiota is a highly complex system. Compositional analysis by DNA sequencing techniques generates a vast amount of data, which are usually high-dimensional, phylogenetically structured, zero-inflated, and over-dispersed^[33]^. These microbial features pose great difficulties when examining microbial communities. Using a suitable method that can better handle such features can improve the interpretation of outcomes. In the following, we discuss the pros and cons of several complementary and sophisticated biostatistical approaches used in the literature to explain microbial relations to host observable traits along the MGBA [Table 2]: (1) constrained methods such as RDA and CCA (acronyms and full names of analytical approaches are listed in Table 3)^[10,34,35]^; (2) RF algorithm^[36-39]^; (3) cluster-based approaches (e.g., the framework of Dirichlet multinomial mixtures^[17,40,41]^ and partitioning-around-medoid algorithm^[42]^); (4) GLMs^[17,43,44]^; and (5) Bayesian linear models^[27]^.

**Table 2:** The pros and cons of analytical approaches used in MGBA research

	**Methods**	**Pros**	**Cons**
Multivariate	Constrained methods	Provide information on explained variation in microbial composition Allow to plot samples, microbial taxa, and observable traits in the same figure Permit follow-up validations of specific taxa	Do not allow to detect complex non-linear relations
	Random forest algorithm	Can identify both linear and non-linear relations Selection of microbial taxa based on their importance is available	Require an appropriate number of samples, which can be determined by out-of-bag error
	Cluster-based approaches	Facilitate comparisons by compressing high-dimensional data	Increased risk of information loss
Univariate	Generalized linear models	Computationally simple and quick	Often limited by a dichotomous outcome (*P* value)
	Bayesian linear models	Present results in the form of a posterior distribution Can increase result precision by including a prior probability distribution Missing observations and multidimensional outcomes are acceptable	Computationally highly demanding

MGBA: Microbiota-gut-brain axis.

**Table 3:** Acronyms and full names of analytical approaches used for exploring microbial relations to host observable traits along the MGBA

**Acronym**	**Full name**
RDA	Redundancy analysis
RF	Random forest
GLM	Generalized linear model
CCA	Canonical correlation analysis
LEfSe	Linear discriminant analysis effect size
MaAsLin2	Microbiome multivariable associations with linear models
ANCOM	Analysis of composition of microbiomes
ALDEx2	ANOVA-like differential expression analysis

MGBA: Microbiota-gut-brain axis.

RDA directly shows how much variation in microbial composition is explained by host observable traits of mental development and health. By drawing a triplot including samples, microbial taxa, and observable traits, we can deduce which taxa fit an RDA model the best and how taxa are potentially related to mental health outcomes. This then provides information for follow-up validations of specific taxa. However, as RDA assumes linear relations between microbial data and observable traits, it is not suitable to explain complex non-linear relations. As a more appropriate alternative, another constrained ordination analysis, i.e., CCA, can be used for analyzing unimodal relations.

Compared to RDA (or CCA) models, RF models can identify both linear and non-linear relations between microbial data and observable traits. However, RF models fit data the best with an appropriate number of samples^[45]^. To determine the best sample size, estimates of out-of-bag error can be used. Out-of-bag estimates reflect the uncertainty of RF models in predicting the outcome of interest with the given sample size^[46]^. When working with an appropriate number of samples, RF models provide useful information regarding the importance of specific microbial taxa, and permit the selection of relevant taxa for downstream validations.

Cluster-based approaches can compress complex high-dimensional microbial data into a simplified low-dimensional matrix and are therefore considered to be a useful tool in identifying microbial patterns with different compositional features. This can largely facilitate the comparisons of mental health outcomes between compositional patterns. However, it is important to note that reduction of dimensionality increases the risk of unexpectedly losing relevant information in the data.

In addition to the three multivariate analytical approaches aforementioned, GLMs and Bayesian linear models can be used to explore univariate relations between single microbial taxa and observable traits^[17,47]^. In general, running a GLM is quicker and computationally less demanding compared to running a Bayesian linear model. However, Bayesian models outperform GLMs in several aspects: (1) use of a posterior distribution as an alternative to a *P* value; (2) ability to incorporate previous information from literature by including a prior probability distribution; and (3) extreme flexibility in straightforwardly fitting models to a complex data set with missing observations and multidimensional outcomes^[48]^. Using these models can help shed light on specific taxa that have the potential of being key biomarkers.

Note that single models may never adequately represent all aspects of the highly complex MGBA. For this reason, an integrative use of analytical approaches in exploring the MGBA in observational studies appears highly advisable. Up till now, an increasing number of techniques have been developed to achieve specific goals in the field of gut microbiota research. One major goal is the identification of microbial taxa that differ in their (relative) abundances between different groups of participants. For this aim, methods such as LEfSe^[49]^, MaAsLin2^[50]^, ANCOM^[51]^, and ALDEx2^[52]^, have been designed. However, the determination of differentially abundant taxa can vary drastically between methods due to varying concepts, algorithms, and requirements, and hence, it is necessary to consider such discrepancies when comparing findings between studies^[53]^. Moreover, due to a recent growing body of longitudinal microbiota cohorts, longitudinal methods have been developed to capture both intra-individual dynamics and inter-individual differences between groups of interest^[54]^. For example, a time-course gene set analysis has been developed and is able to detect changes in a group of genes over time^[55]^. In 2021, Roswall *et al.* implemented this time-course analysis in a longitudinal child cohort and distinguished four microbial developmental trajectories from birth to the age of five years^[56]^. However, to date, longitudinal methods have not been frequently applied to real microbiota data, and their performance awaits to be validated. Summarizing to obtain the most thorough description and information-rich view of the MGBA in observational studies, it is highly recommended to implement multiple complementary and sophisticated statistical approaches.

## MOVING FROM CORRELATION TO CAUSATION

**Figure 1:**
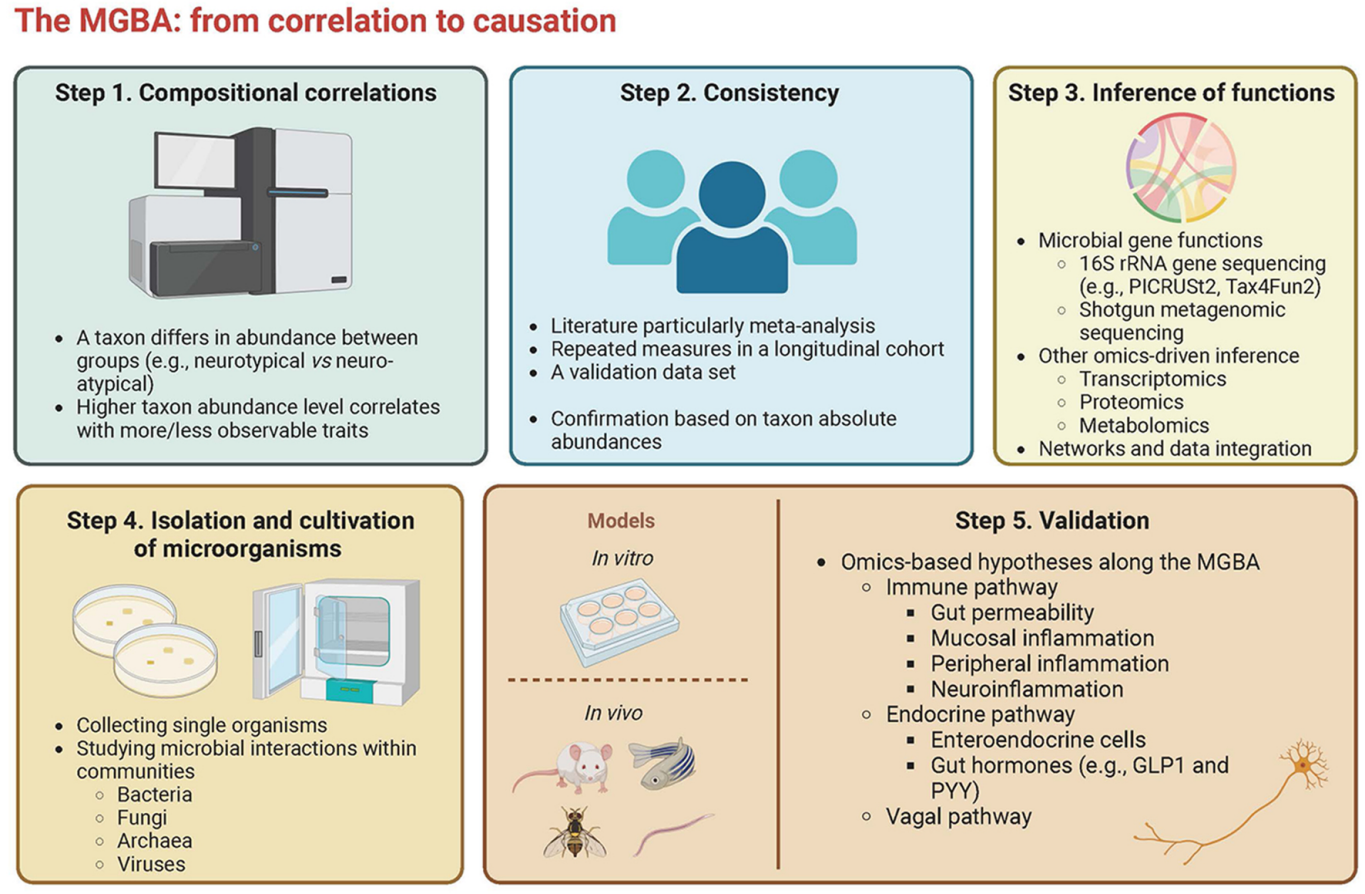
The workflow of moving from sequence-based correlative results towards causality. GLP-1: Glucagon-like peptide 1; MGBA: microbiota-gut-brain axis; PYY: peptide YY. (created with https://biorender.com/)

As the well-known phrase says, “correlation does not imply causation”. It remains a great challenge to translate correlational findings into conclusive proofs of causality, especially along the MGBA. To add more innovative insight into this axis, we introduce a workflow to explore causal relations [Figure 1].

**Step 1** shows two common types of microbial composition-based correlations, including differentially abundant taxa between groups and linear relations between taxa and observable traits. Although these correlations have been reported in an increasing number of studies, little convergence in correlation direction and strength has been reached till now.

**Step 2** introduces several approaches to verify the consistency of the observed correlations. A useful strategy to synthesize diverse outcomes from the literature into a general opinion is meta-analysis. Meta-analyses have been conducted on behavioral profiles on which considerable evidence has been collected to date, such as on ASD^[3]^, MDD^[4,57]^, and ADHD^[2,3]^. Although to the best of our knowledge, meta-analyses have not yet been applied to assessing microbiota-behavior links in community samples due to the limited number of studies, such analyses are highly recommended, once there are sufficient data. When meta-analyses are not feasible, the robustness of results within one study can be enhanced by performing repeated measures or including a validation data set.

Relative abundance-based correlations are widely used in describing microbial links to mental health outcomes. However, relative abundance data have some inherent limitations, including increased correlational biases and false discovery rates^[58,59]^. This may lead to difficulties in objectively capturing inter-individual variations in microbial composition^[60-62]^. Therefore, instead of only using relative abundances, it is recommended to additionally include absolute abundances (or microbial load, measured by, e.g., quantitative PCR or flow cytometry) when attempting to convert statistically significant findings into biological interpretations.

Once the consistency of the observed correlations is verified, we can give more attention to the inference of molecular mechanisms. To this end, an integrative use of omics-driven approaches is suggested in **Step 3**. Microbial gene functions can be the first accessible indicators for explaining the complex gut-brain interplay. Furthermore, although many studies have uncovered aspects of the gut-brain interplay by identifying links to specific microbial taxa, it is of fundamental importance to realize that different taxa can encode the same metabolic functions and play an equivalent role in the gut-brain axis. Therefore, next to focusing on taxonomic variability, functional redundancy (i.e., the capacity to perform the same biochemical function by the coexistence of multiple distinct microbial taxa or their genomes^[63]^) in diverse microbial systems should also be taken into account in MGBA studies. For broadly used 16S rRNA gene sequence data, prediction tools, such as Picrust2, Tax4Fun2, and PanFP^[64-67]^, can leverage the data to the maximum. Although these prediction tools have been criticized for reference bias and limited resolution^[65]^, the increasing availability of reference data renders them more feasible alternatives to the still quite expensive shotgun metagenomic sequencing. Additionally, other omic techniques can be incorporated into the selection process of key pathways and possible biomarkers through multi-omics data integration approaches, such as iClusterPlus, mixOmixs, JIVE, and PARADIGM^[68]^: (1) transcriptomics provides information on sample-specific gene expression features (tools include, e.g., DESeq2, edgeR, and limma^[69-71]^); (2) proteomics measures the entire set of proteins in samples and therefore can be used to discover potential biomarkers (tools include, e.g., MaxQuant, SpectroNaut, PEAKS, and DIA-NN^[72-75]^); and (3) metabolomics studies metabolites in samples (tools include, e.g., Mzmine3, MetaboAnalyst 5.0, and MetFlow^[76-78]^). These techniques can help increase the understanding of relevant molecular pathways active and relevant in specific conditions^[79]^.

Before validating the inferred molecular mechanisms of candidate taxa, **Step 4** emphasizes the importance of isolation, cultivation, and characterization of specific microorganisms and even whole microbial communities. The availability of cultured representatives of target microorganisms is a prerequisite to meet the demand for experimental designs and even therapeutic strategies. A study in 2005 reported that approximately 80% of human gut bacteria have not been cultured yet^[80]^. Compared with labor-intensive and low-throughput traditional cultivation approaches, new cultivation tools such as different droplet-based platforms, enable the anaerobic growth of microbial cells in millions of microscale droplets^[81,82]^. These picoliter droplets can be automatically separated based on colony density to enhance the expansion of slow-growing cells^[82]^. With high-throughput cultivation approaches being developed rapidly, it will be technically possible in the coming decades to produce personalized collections of gut microbial taxa with known genotypical and phenotypical characteristics^[83]^.

In addition to collecting single microorganisms, it is also important to intensify research on studying interactions between various microorganisms: not only predominant bacteria, but also other microbes, such as fungi and archaea, as well as viruses^[83]^. Fungi regulate gut immunity and are involved in gut-related diseases, such as inflammatory bowel disease, irritable bowel syndrome, and colorectal cancer^[84]^. After millions of years of coevolution, gut fungi and bacteria have developed various types of interactions, including mutualistic, commensal, and competitive relations^[84]^. Archaea in the human gut, mainly composed of methanogens, produce methane (i.e., a potential neuromodulator and immunoregulator) and affect host gut motility^[85]^. Archaea also interact with bacteria in the gut by utilizing bacteria-derived products and consuming hydrogen, which improves energy yield and shifts metabolic outcomes^[85]^. Moreover, the binding of viruses to bacteria has been shown to promote bacterial adhesion to eukaryotic cells and to increase coinfection and genetic recombination^[86]^. These complex interactions between host, bacteria, fungi, archaea, and viruses constitute important challenges, but also underline the value of efforts aimed at obtaining a more detailed picture of these dynamic interactions. Only then will we be able to determine more precisely how different microorganisms influence host phenotypes.

**Step 5** presents currently available *in vitro* and *in vivo* models used in validating pathways (e.g., immunity, endocrine system, and vagus nerve as three main pathways) along the MGBA^[1,11]^. Depending on study designs, different *in vitro* and *in vivo* models can be selected, such as organoids and animals (e.g., rodents, zebrafish, fruit flies, and nematodes), respectively^[1,87,88]^. Although *in vitro* models are low-cost, time-efficient, and highly repeatable compared to *in vivo* models, they are often not performed in physiological conditions and hence lack precise descriptions of underlying molecular mechanisms. In spite of this, newly developed *in vitro* models increasingly address these disadvantages. For instance, organoids are self-organized three-dimensional tissue constructs that show *in vivo*-like structure and regional specification^[89]^. Additionally, *in vivo* rodent models mimic potential causes and phenotypic outcomes of certain mental disorders (e.g., ASD, and depression and anxiety disorders), adding invaluable credits to causality exploration^[87,90]^. For example, the probiotic *Limosilactobacillus reuteri* (previously called *Lactobacillus reuteri*) was applied to specific-gene mutant rodent models with behavioral deficits, and this taxon rescued social deficits and improved oxytocin levels^[87]^. According to the FAO/WHO definition, a probiotic strain must be (1) sufficiently characterized; (2) safe for the intended purpose; (3) supported by at least one human clinical study; and (4) alive at an adequate amount during shelf life^[91]^. Although not all candidate taxa may seem qualified probiotics, their metabolites may be interesting biomarkers or even drugs for various mental disorders.

Despite the feasibility given by model organisms in exploring causality, it is important to reiterate that host observable traits are often different between model organisms and human beings, which to some extent impedes the translation from bench to bedside^[1]^. For this reason, well-established validation standards must be applied to animal studies beforehand^[11]^. Moreover, it has to be noted that the gut microbiota is a highly complex and interactive consortium, and studies of this community should not be restricted to specific microorganisms. To explore microbial communities as a whole, fecal microbiota transplantation (i.e., procedures that transfer stool-whole microbial communities from a donor to a recipient) can provide a more panoramic view of causal relations along the MGBA^[92]^. Recently, authoritative guidance (i.e., Guidelines for Reporting Animal Fecal Transplant) has been developed for preclinical fecal microbiota transplant, which will further facilitate the replicability and reproducibility of studies focused on causality^[93]^. Nevertheless, how microbes interact with each other and jointly influence host phenotypes at a molecular level awaits to be fully understood. This is an essential part of the puzzle that should receive more attention over the coming years.

## CONCLUSIONS

Observational studies have uncovered a large number of correlations between gut microbiota composition and host mental development and health. However, these findings often lack consistency, impeding biological understanding and mechanistic verifications. To inspire future MGBA research, we (1) present several considerations for confounder selection, including the use of DAGs, discontinuing the overdependence on *P* values, checking for and reporting collinearity, presenting results adjusted and not adjusted for confounders, and pre-registering studies and analytical methods on open platforms; (2) recommend the use of a group of complementary and sophisticated biostatistical approaches when deciphering the complexity of the microbiota-host relations; and (3) introduce a five-step workflow for shifting sequence-based correlative results into more causal conclusions, including the identification of the microbiota-host correlations, the verification of the consistency of the observed correlations, the inference of microbial functions via different approaches, the isolation and cultivation of interesting microbial taxa, and the mechanistic validation on these taxa in *in vitro* and *in vivo* models. At this highly exploratory stage of the MGBA field, the first priority is to carry out bias-controlled replication studies to reach a consensus on the type and direction of associations. Once consistency is determined, more attention can be given to causality.

## DECLARATIONS

### Copyright

© The Author(s) 2023.
